# Analysis of *Pseudomonas aeruginosa* biofilm membrane vesicles supports multiple mechanisms of biogenesis

**DOI:** 10.1371/journal.pone.0212275

**Published:** 2019-02-14

**Authors:** Adam C. Cooke, Alexander V. Nello, Robert K. Ernst, Jeffrey W. Schertzer

**Affiliations:** 1 Department of Biological Sciences, Binghamton University, Binghamton, New York, United States of America; 2 Binghamton Biofilm Research Center, Binghamton University, Binghamton, New York, United States of America; 3 Department of Microbial Pathogenesis, School of Dentistry, University of Maryland, Baltimore, Maryland, United States of America; Universidad Nacional de la Plata, ARGENTINA

## Abstract

Outer Membrane Vesicles (OMVs) are ubiquitous in bacterial environments and enable interactions within and between species. OMVs are observed in lab-grown and environmental biofilms, but our understanding of their function comes primarily from planktonic studies. Planktonic OMVs assist in toxin delivery, cell-cell communication, horizontal gene transfer, small RNA trafficking, and immune system evasion. Previous studies reported differences in size and proteomic cargo between planktonic and agar plate biofilm OMVs, suggesting possible differences in function between OMV types. In *Pseudomonas aeruginosa* interstitial biofilms, extracellular vesicles were reported to arise through cell lysis, in contrast to planktonic OMV biogenesis that involves the Pseudomonas Quinolone Signal (PQS) without appreciable autolysis. Differences in biogenesis mechanism could provide a rationale for observed differences in OMV characteristics between systems. Using nanoparticle tracking, we found that *P*. *aeruginosa* PAO1 planktonic and biofilm OMVs had similar characteristics. However, *P*. *aeruginosa* PA14 OMVs were smaller, with planktonic OMVs also being smaller than their biofilm counterparts. Large differences in *Staphylococcus* killing ability were measured between OMVs from different strains, and a smaller within-strain difference was recorded between PA14 planktonic and biofilm OMVs. Across all conditions, the predatory ability of OMVs negatively correlated with their size. To address biogenesis mechanism, we analyzed vesicles from wild type and *pqsA* mutant biofilms. This showed that PQS is required for physiological-scale production of biofilm OMVs, and time-course analysis confirmed that PQS production precedes OMV production as it does in planktonic cultures. However, a small sub-population of vesicles was detected in *pqsA* mutant biofilms whose size distribution more resembled sonicated cell debris than wild type OMVs. These results support the idea that, while a small and unique population of vesicles in *P*. *aeruginosa* biofilms may result from cell lysis, the PQS-induced mechanism is required to generate the majority of OMVs produced by wild type communities.

## Introduction

The biofilm mode of growth predominates in natural [1] and disease [2] environments, with estimates of greater than 65 percent of infections being biofilm-related [3]. Planktonic bacteria transition to a biofilm lifestyle by first attaching to a surface [4,5]. Afterwards, they produce an extracellular polymeric substance (EPS) that encases the bacteria, and protects them from the environment [6]. The EPS is composed of polysaccharides [7], extracellular DNA [7,8], proteins [7,9], and lipids [9] that can be found in the form of OMVs [10]. Recent literature suggests that OMV production may contribute to biofilm formation in *Helicobacter pylori* [11], *Vibrio cholerae* [12], and *Pseudomonas putida* [13], and inhibit biofilm formation in *Xylella fastidiosa* [14]. Together with the known ubiquity of OMV production among Gram-negative species [15], this suggests that OMVs may carry out important functions for organisms living the biofilm lifestyle.

Most studies that have assessed OMV structure and function have used vesicles isolated from planktonic culture. From these studies, a variety of functions have been ascribed to OMVs (reviewed in [15,16]). They act as packages to transport toxins [17–25], plasmid DNA [26], small RNAs [27,28], and quorum signaling molecules [19] between cells. Because OMVs resemble the cells from which they arise [29], they can bind and sequester antibodies and bacteriophage away from the parent bacteria [30,31], and they can also use packaged enzyme activities to degrade antibiotics in the environment [32,33]. Because all these studies were performed using planktonic OMVs, it remains unclear whether any or all of these functions can also be ascribed to biofilm OMVs. Proteomic profiling has revealed that the protein complement of *P*. *aeruginosa* planktonic OMVs and biofilm OMVs differ [10,34–37], and it has been reported that physical characteristics of the vesicles themselves can deviate between planktonic and biofilm OMVs [10].

Several mechanisms have been proposed to describe the process of OMV biogenesis in individual species [15,38–45] but none has been generally adopted across species and environments. A recent study found that membrane vesicle (MV) biogenesis in *P*. *aeruginosa* interstitial biofilms was due to cell lysis via the RecA-mediated SOS response [40]. Following lysis, membrane debris re-circularized to form vesicles that randomly captured the contents recently released from the exploded cells [40]. This mechanism of biogenesis is quite different from what occurs in unstressed planktonic *P*. *aeruginosa* cultures [19,46]. Under these conditions, the *Pseudomonas* Quinolone Signal (PQS) induces outer membrane curvature [19,39], which leads to OMV biogenesis without the involvement of cell lysis [46–48]. Planktonic OMV biogenesis in several other species was also found to occur without cell lysis [24,49,50]. However, a difference in the mechanism of vesicle biogenesis between planktonic and biofilm populations could explain observed differences in OMV proteomic content and physical characteristics. If cell lysis is the primary driver of biofilm MV production, one might expect to find greater variability in protein content, size and physical properties since such vesicles would be formed by random recircularization of membrane fragments rather than a controlled bacterial process.

Despite the importance of the biofilm mode of growth and the established abundance of OMVs within the biofilm matrix, little work has been done to investigate the physical characteristics, function, and biogenesis of OMVs during biofilm growth. The goal of this work was to compare OMVs harvested from planktonic cultures to those found in agar plate biofilms. This biofilm model was chosen because it was previously used to establish benchmarks for biofilm OMV size and characteristics [10,51] and it has been the preferred system for generating biofilm OMVs for proteomic analysis [34,35,37]. We used Nanoparticle Tracking Analysis (NTA) to analyze fully hydrated and minimally processed OMV samples from several strains and also compared function by assessing predatory ability against OMV-susceptible *Staphylococcus epidermidis* [19]. Finally, we investigated whether *P*. *aeruginosa* biofilm OMVs were produced using the same mechanism as planktonic OMVs by assessing the role of PQS in their biogenesis. Using these tools we identified similarities and differences between planktonic and biofilm OMVs, while providing evidence that multiple mechanisms of MV biogenesis exist that may predominate under different conditions.

## Materials and methods

### Bacterial strains and media

Strains and plasmids used are listed in Table 1. For the generation of mutants, *P*. *aeruginosa* and *Escherichia coli* were routinely cultured in or on Lysogenic Miller broth/agar (Fisher Scientific). For all other purposes, *P*. *aeruginosa* was cultured on tryptic soy broth (TSB) or tryptic soy agar (TSA) (BD Biosciences). *S*. *epidermidis* was cultured on brain heart infusion broth or brain heart infusion agar. When necessary, media were supplemented with gentamicin (Amresco) at 20 μg/ml for *E*. *coli* and 50 μg/ml for *P*. *aeruginosa*.

**Table 1:** Strains used in this study.

Strain or Plasmid	Description	Source or Reference
**Strains:**		
*E*. *coli*		
DH5α	F– Φ80lacZΔM15 Δ(lacZYA-argF) U169 recA1 endA1 hsdR17 (rK–, mK+) phoA supE44 λ– thi-1 gyrA96 relA1	[52]
*P*. *aeruginosa*		
PAO1	Wild-type *Pseudomonas aeruginosa* strain	[53]
PA14	Wild-type *Pseudomonas aeruginosa* strain	[54]
Δ*pqsA*	*pqsA* clean deletion in PA14 background	Kind gift of Marvin Whiteley
*S*. *epidermidis*		
1457	Wild-type *Staphylococcus epidermidis* strain	[55]
**Plasmids**		
pJN105	Gm^R^; *araC-pBAD* expression vector	[56]
pJN105-*pqsA*	Gm^R^; pJN105-derived *pqsA* overexpression vector	This study

### DNA manipulations

Restriction endonucleases and T4 DNA ligase were purchased from New England Biolabs. Chromosomal DNA was isolated from *P*. *aeruginosa* using DNeasy Blood & Tissue Kits (Qiagen). Plasmid DNA was isolated, and DNA fragments were purified using EconoSpin spin columns for DNA (Epoch Life Science). DNA was amplified using Platinum *Taq* Polymerase High Fidelity (Invitrogen).

### Generation of the *pqsA* overexpression mutant

The *pqsA* gene was amplified from PA14 chromosomal DNA using the primers listed in S1 Table in the supplemental material. The PCR product was purified, digested using restriction endonucleases PstI and SacI, and ligated into the pJN105 plasmid digested with the same endonucleases. The plasmid was transformed into *E*. *coli* strain DH5α to maintain the plasmid, and the sequence of the insert was verified using DNA sequencing. The primers used for sequencing the insert are also listed in S1 Table. The plasmid was subsequently purified from *E*. *coli* DH5α and electroporated into the PA14 *pqsA* mutant strain using a method described previously [57]. The electroporated cells were grown on LB agar containing gentamicin to select for the plasmid.

### Isolation of OMVs from planktonic cultures

Cultures were grown in TSB at a starting OD_600_ of 0.01 and grown in 50 ml cultures at 37°C with shaking at 250 r.p.m. until early stationary phase. Cells were centrifuged at 16,000×g for 10 minutes at 4°C, the supernatant was transferred, and cells were centrifuged again at 16,000×g for an additional 10 minutes at 4°C to remove the majority of the cells from the supernatant. All low speed centrifugations (below 20,000 × g) were performed using a ThermoFisher Fiberlite F15s-8x50c rotor. The supernatant was then passed through a 0.45 μm polyethersulfone (PES) filter, and OMVs were pelleted at 150,000×g and 4°C for 75 minutes in a Thermo Scientific S50-A fixed angle rotor (equivalent to [58]). The pellet formed was then resuspended in 0.22 micron filter sterilized MV buffer (50 mM Tris, 5 mM NaCl, 1 mM MgSO_4_, pH 7.4) [59] and the suspension was centrifuged again for 75 minutes at 150,000×g and 4°C. After the second centrifugation, the pellet was then resuspended in 1 ml of MV buffer.

### Isolation of OMVs from agar plate biofilms

Biofilms were inoculated and grown as described previously [10]. Briefly, petri dishes with a diameter of 90 mm containing 25 ml of TSA were inoculated by pipetting 1–2 ml of overnight culture onto the plate, swirling the plate to coat all available surface area with culture, and pipetting off the excess. The biofilms were then grown at 37°C for 24 hours and harvested into 0.85% saline using a cell scraper, unless otherwise noted. OMVs were liberated from the biofilm either by vortexing or homogenization. Vortexing was performed as described previously [10]. Briefly, the removed biofilm was vortexed in saline for 3 minutes, then centrifuged for 20 minutes at 12,000×g to pellet the cells. Afterwards, the supernatant was retained, the pellet was resuspended in 0.85% saline, and the process was repeated three times. The retained supernatants were then pooled together and centrifuged once more at 12,000×g to pellet the few remaining cells, followed by filtration though a 0.45-micron PES filter. Alternatively, homogenization was equally effective at liberating OMVs that were indistinguishable in size to those harvested by vortexing (S1 Fig), while also avoiding any detectable cell lysis (S2 Fig). OMVs were liberated from samples by homogenizing the biofilms with a Tissue Master 125 homogenizer (OMNI International) in 0.85% saline at maximum power for 10 seconds. Afterwards, the samples were centrifuged at 16,000×g for 10 minutes, and the supernatant was passed through a 0.45-micron filter. From this step, the supernatants were filtered and ultracentrifuged as described above for the isolation of planktonic OMVs.

### Quantification of OMV size and concentration

The Malvern NanoSight NS300 was used to quantify the concentration and the diameters of the OMVs using single nanoparticle tracking. For each sample, three independent 30 second videos of the vesicles were recorded at a temperature of 25°C using the accompanying NTA software at a camera level setting of 12. The Brownian motion of each individual vesicle was determined by the software which then converted this value to the hydrodynamic diameter of each vesicle using the Stokes-Einstein equation. A detection threshold level of 38 was used to limit the amount of background noise. Vesicle samples were diluted prior to using the NanoSight such that the final number of OMVs per frame would be between 20 and 100, as to not oversaturate the flow chamber. The concentration was found by using the volume of the flow cell imaged by the sCMOS camera on the NanoSight to convert the average number of particles per frame for a sample to the number of particles per ml, and then multiplying by the dilution factor.

### Lysis identification assay

To determine whether homogenization resulted in cell lysis, planktonic cultures were grown to late-exponential phase. Cells were washed in 0.85% saline, pelleted, and resuspended in MV buffer to an OD_600_ of 5. Pelleting was performed at 16,000×g for 10 minutes at 4°C. Cells were then homogenized for 0, 5, 10, 15, or 20 seconds, or were lysed using sonication. Afterwards, cells and cell debris were pelleted as described above, and 2μl of supernatant from each sample was used to test for the presence of succinate dehydrogenase (SDH) using a previously described assay [46,60]. Briefly, the supernatant was added to a reaction mixture totaling 200μl, and consisting of 50mM Tris-HCl, pH 8.5, 4mM KCN, 0.04mM DCPIP, 0.2mM PMS, 40mM disodium succinate. The reaction took place in a 96 well plate, and the Abs_600_ was measured for 5 minutes using a Tecan Infinite M200 Pro microplate reader. To test for SDH activity in homogenized agar plate model biofilms, PAO1 and PA14 biofilms grown for 24 hours as described above were harvested into ice-cold MV buffer and homogenized for 10 seconds or lysed using sonication. Cells and large membrane fragments were then pelleted at 16,000×g for 10 minutes at 4°C, and 2μl of supernatant from each sample was used to test for the presence of succinate dehydrogenase, as described above. To test for SDH activity in biofilm vesicles, supernatant from homogenized and lysed biofilms were harvested as described above, filtered through a 0.45-micron filter, and ultracentrifuged for 75 minutes at 150,000×g to concentrate the vesicles. Pellets from homogenized biofilms were then resuspended in 200 μl of MV buffer whereas lysed biofilm cell fragments were resuspended in 500 μl of MV buffer, and the suspensions were tested for SDH as described above.

### Quantification of PQS

PQS extraction was performed based on previously published protocols [59,61]. Briefly, PQS was extracted from homogenized biofilms 1:1 with acidified ethyl acetate (0.1 ml per liter glacial acetic acid). The organic phase was removed and dried under nitrogen gas. The organic material was resuspended in optima grade methanol (Fisher), and PQS was separated using thin layer chromatography. The 10x20 cm silicone TLC plate (Millipore) used was impregnated with ACS reagent grade potassium phosphate and activated at 100°C for 1 h. PQS was visualized on the TLC plate using long wave UV light to excite the compound. Densitometry was performed by comparing intensity of the sample to 100, 200, 300, 400, and 500 μM standards (UVP VisionWorks LS).

### Colony forming unit quantification in agar plate model biofilms

Following homogenization of the biofilms that were harvested at 4-hour intervals, serial dilutions were made in saline, and dilutions were plated on TSA. CFU counts were performed after the plates were incubated overnight at 37°C.

### OMV agar growth inhibition assay

OMVs isolated via ultracentrifugation were passed through sterile 0.45 μm PES filters to ensure sterility of the OMVs. Prior to testing, the number of OMVs in each sample was determined by diluting 10 μl aliquots of OMVs in MV buffer, and quantifying via nanoparticle tracking as described above. Each sample was then diluted with sterile MV buffer to 1x10^10^ OMVs/ml. To test inhibitory effects of the OMVs, *S*. *epidermidis* was seeded onto 90mm diameter petri dishes containing 25 ml of BHI each using a top agar method for a more even dispersal of the bacteria. Exponential phase *S*. *epidermidis* was added to 3 ml of top agar (0.7% agar in BHI) kept at 50°C to an OD_600_ of 0.1. The top agar was then poured onto the petri dish which was swirled to allow for even coverage of the top agar on the plate. Sterile cotton disks with a diameter of 6 mm were placed onto the cooled top agar, and 10 μl of 25 μg/ml gentamicin, MV buffer, or 1x10^10^ OMVs/ml of vesicles in MV buffer were pipetted onto the disks. As a control, we tested whether the LPS content of the OMVs had any effect in the growth inhibition assay. Using the same disk diffusion technique, *S*. *epidermidis* lawns were exposed to the estimated amount of LPS in 1×10^8^ OMVs (see below), as well as 5 times this concentration. Plates were incubated at 37°C for 16 hours, and the zones of clearing were measured by subtracting the diameter of the cotton discs from the diameter of the zone of inhibition. In addition, 100μl of each sterilized OMV sample was spread onto TSA plates and incubated at 37°C for 24 hours to confirm sterility. All data obtained from vesicle samples that showed evidence of contamination were discarded.

### Lipopolysaccharide extraction and preparation

**Fig 1:**
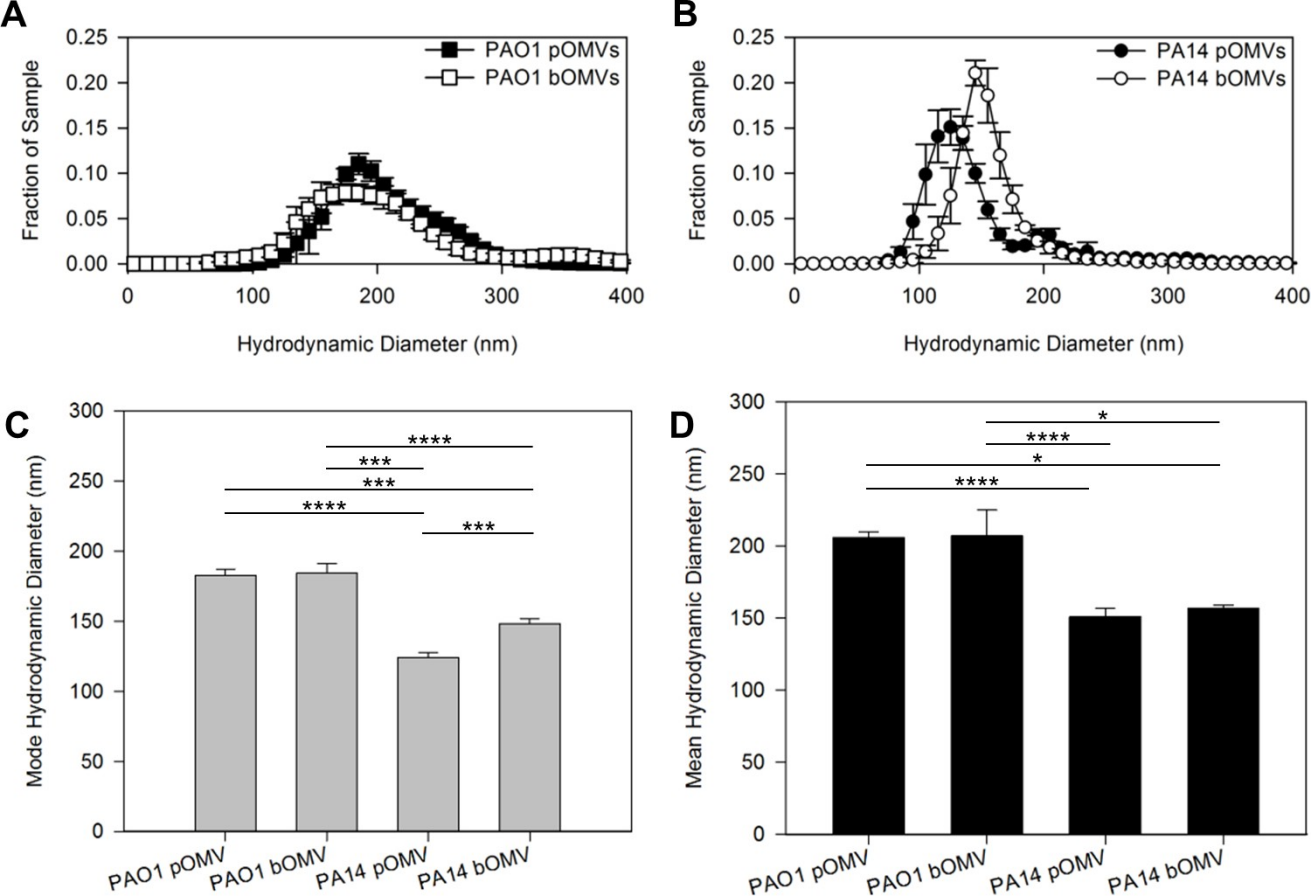
Size of *P*. *aeruginosa* OMVs isolated from biofilms and planktonic cultures. Size distributions (A and B), as well as mode (C) and mean (D) hydrodynamic diameters of the OMVs were generated using nanoparticle tracking for OMV samples isolated from biofilms and planktonic cultures of *P*. *aeruginosa* PAO1 (A, C and D) and PA14 (B-D). Error bars represent standard error. Statistical significance was analyzed by two-tailed Student t-test (* p < .05; ***p < .005; ****p < .001). n≥4.

Large-scale *P*. *aeruginosa* LPS preparations were isolated using a hot phenol/water extraction method after growth in lysogenic broth (LB) supplemented with 1 mM MgCl2 at 37°C [62]. Subsequently, LPS was treated with RNase A, DNase I and proteinase K to ensure purity from contaminating nucleic acids and proteins [63]. Individual LPS samples were additionally extracted to remove contaminating phospholipids [64] and TLR2 contaminating proteins [65]. Finally, individual LPS preparations were resuspended in 500 μl of water, frozen on dry ice and lyophilized. To estimate the amount of LPS in each vesicle for PAO1 and PA14, we assumed the concentration of LPS within the outer membrane to be 1.75 attamol/μm^2^ and multiplied this by the surface area of the vesicles (0.11 μm^2^ per OMV for PAO1, 0.071 μm^2^ per OMV for PA14). Surface areas were calculated using the diameters of planktonic PAO1 and PA14 OMVs determined by NTA (Fig 1). Using these values, we determined the concentration of LPS that would be equivalent to 1×10^10^ OMVs/ml for PAO1 to be 8.09 μg/ml, and for PA14 to be 5.22 μg/ml. 10 uL from these LPS stock solutions (as well as from 5x-concentrated stocks of each) were pipetted onto filter disks that were placed onto *S*. *epidermidis* growth plates as described above for the growth inhibition assay.

## Results

### OMV size differs more between strains than between growth modes

We first compared OMV size differences between agar plate model biofilms and planktonic cultures of *P*. *aeruginosa* strains PAO1 and PA14. In contrast to results previously reported using electron microscopy [10], we found that differences in OMV size between biofilms and planktonic cultures were minimal for PAO1 when measured using NTA (Fig 1A, 1C and 1D). PAO1 planktonic OMVs had a mode diameter of 182.4 ± 4.9 nm and an average diameter of 206.0 ± 4.1nm, whereas PAO1 biofilm OMVs had a mode diameter of 184.3 ± 6.9 nm and an average diameter of 207.5 ± 17.6nm. We also compared OMV size in PA14 and found that planktonic OMVs had a mode diameter of 124.2 ± 3.8 nm and an average diameter of 151.0 ± 5.7nm, whereas biofilm OMVs had a mode diameter of 148.5 ± 3.5nm an average diameter of 156.8 ± 2.5nm (Fig 1B–1D). While the average OMV size did not differ significantly between planktonic OMVs and biofilm OMVs in PA14, the modes sizes suggest that the planktonic OMVs were smaller than the biofilm OMVs, as does the difference in size distribution of the two populations (Fig 1B). Furthermore, we observed that PAO1 OMVs were larger than PA14 OMVs under all conditions, whether measured by mode diameter (Fig 1C) or mean diameter (Fig 1D). These data suggest that OMV size characteristics of *P*. *aeruginosa* OMVs are dependent on the strain producing the OMVs, and the environment in which the bacteria are producing the OMVs.

### Predation of *P*. *aeruginosa* OMVs is dependent on strain and growth mode

**Fig 2:**
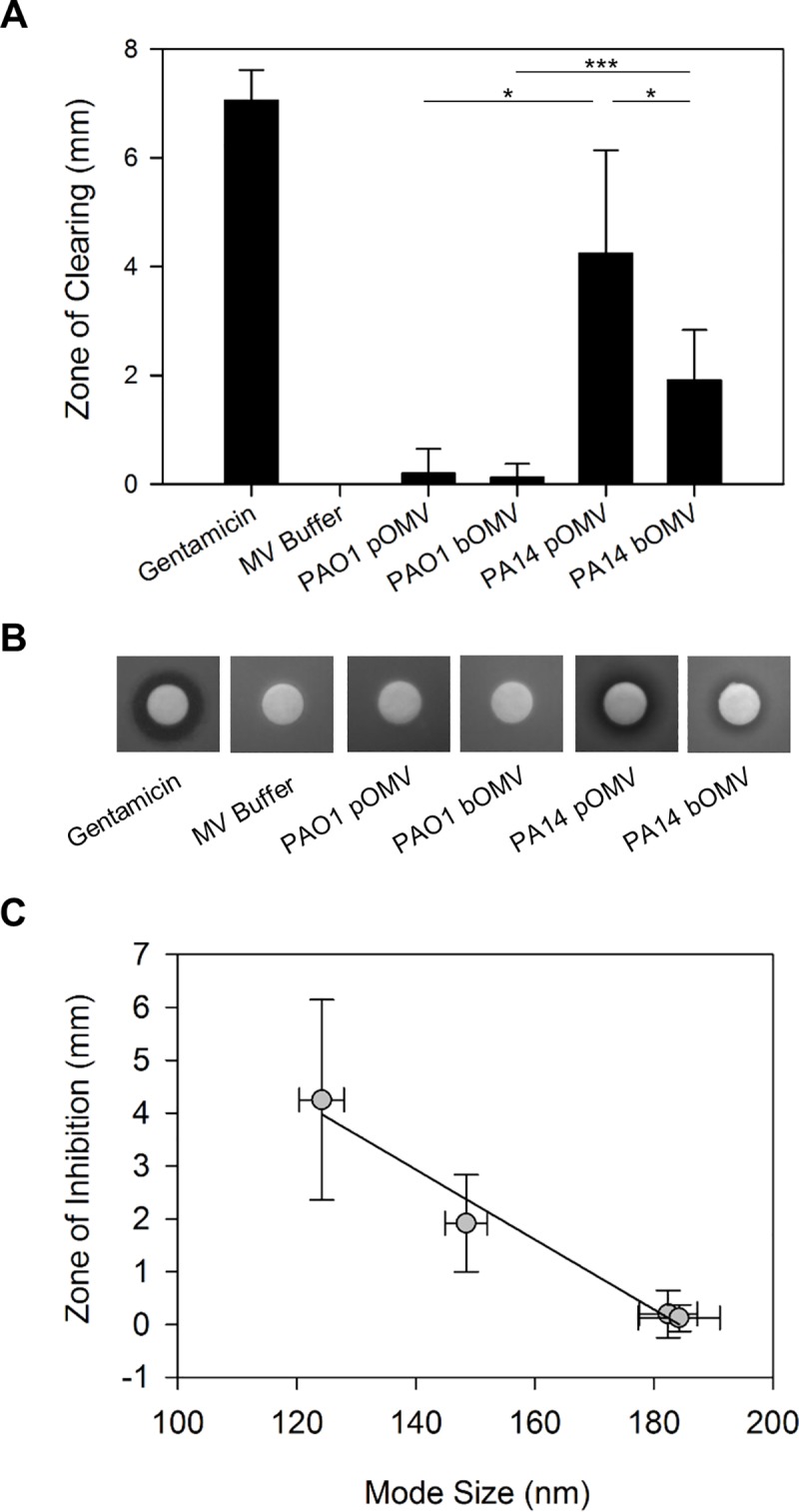
Predatory activity of *P*. *aeruginosa* OMVs against *S*. *epidermidis*. Disk diffusion assays were performed in triplicate using 250 ng of gentamicin as a positive control, MV buffer as a negative control, and 1x10^8^ OMVs in MV buffer from biofilms and planktonic cultures of PAO1 and PA14 (A). Images showing representative zones of clearing are also included (B). Panel (C) shows the relationship between zone of inhibition and OMV size (R^2^ = 0.9733). Error bars for the zones of inhibition represent standard deviation. Error bars for mode sizes represent standard error. Statistical significance was analyzed by two-tailed Student t-test (*p < .05; ***p < .005). n≥4.

After finding differences in size between OMVs, it was of interest to investigate whether these different OMVs had differences in function. We assessed the ability of the purified *P*. *aeruginosa* OMVs to inhibit growth of *S*. *epidermidis* in a disk-diffusion assay as a measure of their predatory ability [17,18]. OMVs (1 x 10^8^, quantified by NTA) were spotted onto cotton disks and observed for their ability to inhibit growth. PAO1 OMVs were largely ineffective at producing zones of clearing, with biofilm OMVs creating an average zone of clearing of 0.12 ± 0.26 mm, and planktonic OMVs creating an average zone of clearing of 0.20 ± 0.44 mm (Fig 2A and 2B). PA14 OMVs produced larger zones of clearing regardless of source: 1.92 ± 0.92 mm for biofilm OMVs and 4.26 ± 1.90 mm for planktonic OMVs (Fig 2A and 2B). While we saw no significant differences between planktonic and biofilm OMVs in PAO1, we did see a significant difference in predation between planktonic and biofilm OMVs in PA14 (Fig 2A). As a control, we extracted LPS from both our PAO1 and PA14 strains and spotted these samples in our disk-diffusion assay, as was done for OMVs (see methods). No zone of clearing was measured in either case, confirming that the lipid content of the OMVs was not responsible for their ability to inhibit growth (S3 Fig). Interestingly, when we compared the growth inhibition results to the OMV size distributions measured by NTA in Fig 1, we saw that the smaller PA14 OMVs were more effective at inhibiting growth of *S*. *epidermidis* than the larger PAO1 OMVs. Looking only at PA14 OMVs, the smaller planktonic OMVs were more effective than the larger biofilm OMVs. These observations suggest that differences in OMV predatory ability vary more between strains than between modes of growth for an individual strain. Across all cases, however, there was a strong inverse correlation between killing effectiveness and mode OMV size (Fig 2C).

### OMV production in *P*. *aeruginosa* biofilms is PQS-Dependent

**Fig 3:**
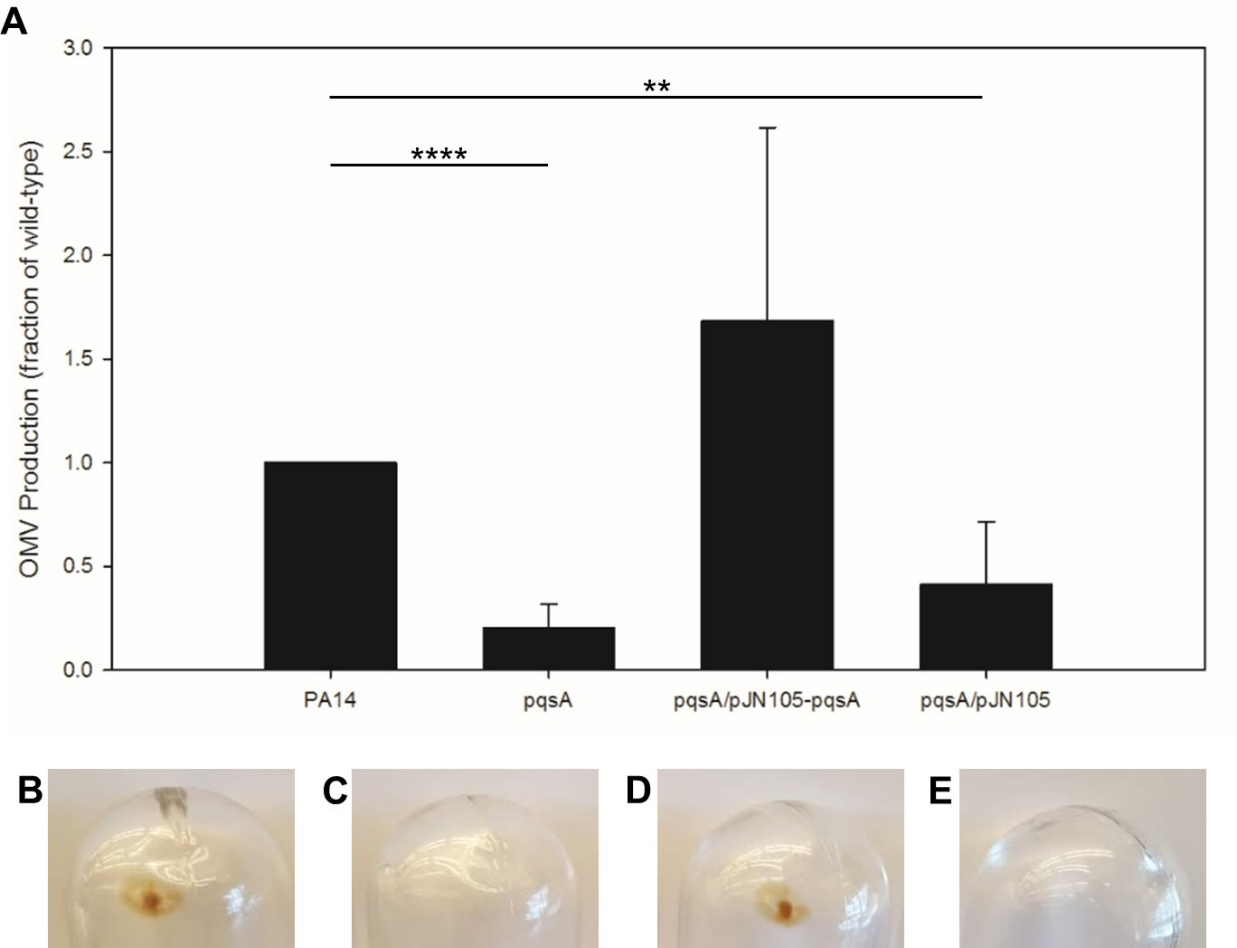
OMV production in PQS biosynthetic mutant biofilms. OMV concentration was quantified using NTA (A). The images show ultracentrifuge pellets representative of the wild type (B), the *pqsA* mutant (C), the complemented *pqsA* mutant (D), and the *pqsA* mutant carrying the empty vector (E). All strains containing plasmids were grown in the presence of gentamicin at 50 μg/ml. Error bars represent standard deviation. Statistical significance was analyzed by two-tailed Student t-test (** p < .01; ****p < .001). n = 7.

To address the question of whether OMV biogenesis proceeds by different mechanisms in biofilms *versus* planktonic cultures, we began by investigating whether biofilm OMV production was dependent upon the production of PQS. Induction of OMV biogenesis by PQS is a well-established mechanism in planktonic cultures [19,39,46,66,67], but it has not been well characterized in biofilms. Because membrane vesicles produced in the absence of PQS may be of variable composition, including both inner and outer membrane [68], we will refer to them by the general terms “vesicle” or “MV” to distinguish them from OMVs. To study MV biogenesis in the absence of PQS, we grew *pqsA* mutant biofilms, as the PqsA gene product is required for the synthesis of PQS [69]. We saw that the *pqsA* mutant produced 5-fold fewer MVs than the wild type, and that complementation of the mutant *in trans* restored OMV production to wild type levels (Fig 3A). These data were supported by the observation of noticeable OMV ultracentrifuge pellets in the wild-type (Fig 3B) and complemented strain (Fig 3D). MV pellets in the *pqsA* mutant (Fig 3C) and the empty vector control (Fig 3E) were difficult to detect visually. These results suggest that PQS plays an important role in OMV biogenesis in the agar plate biofilm model, as it also does in planktonic OMV biogenesis.

### OMV production follows PQS production in *P*. *aeruginosa* biofilms

**Fig 4:**
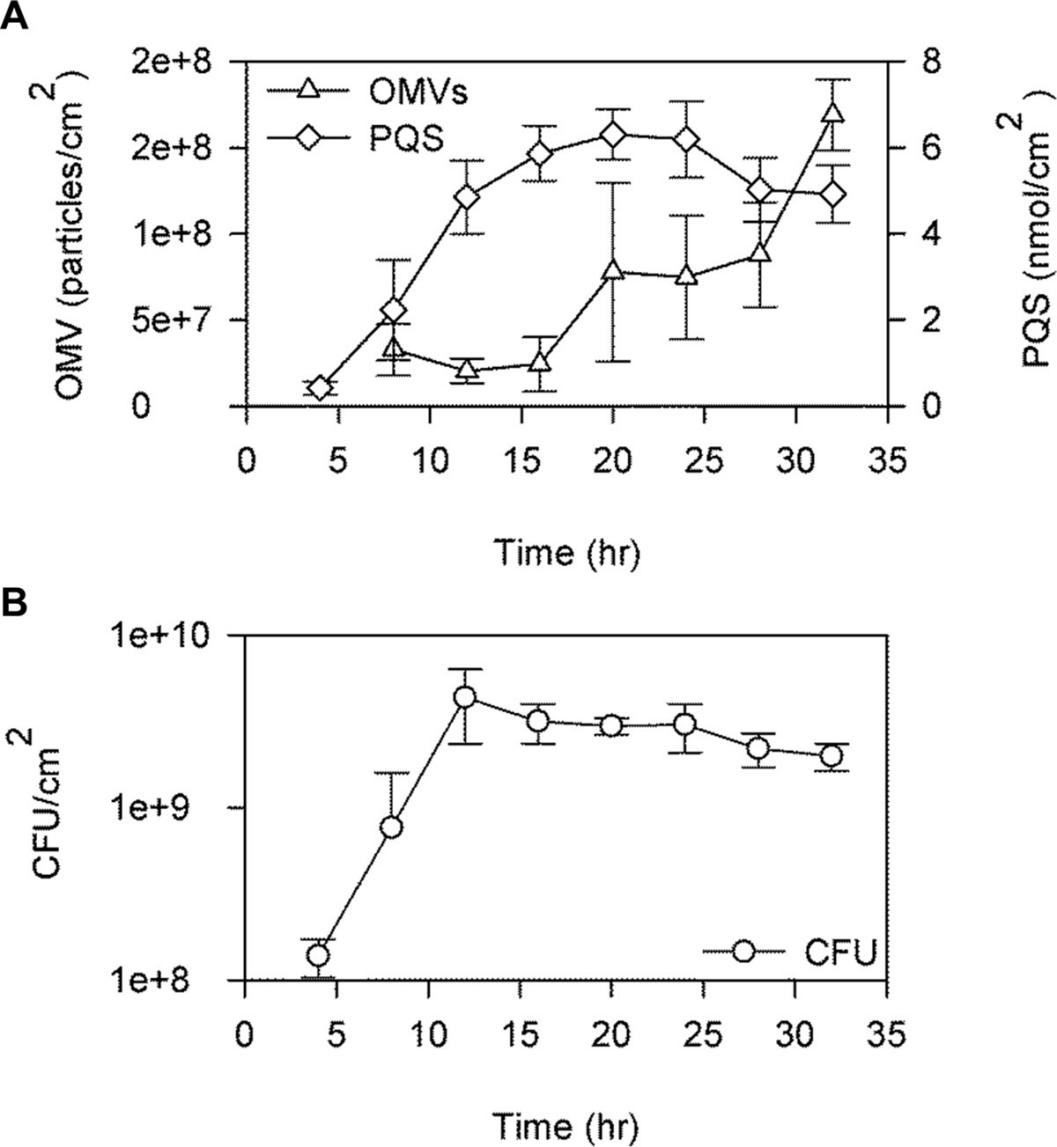
Production of PQS and OMVs in *P*. *aeruginosa* PA14 biofilms over time. The number of OMVs and amount of PQS per cm^2^ (A), as well as the number of CFUs per cm^2^ (B) in PA14 biofilms were determined every 4 hours via nanoparticle tracking, thin layer chromatography (TLC), and serial dilution followed by plating, respectively. Error bars represent standard deviation. n = 3.

Since biofilm OMV production was observed to be dependent on the presence of *pqsA*, we investigated whether PQS concentration and OMV concentration increase as the number of cells in the population increases, as they do in planktonic cultures [19,46,70]. We analyzed OMV and PQS concentrations in PA14 biofilms every 4 hours for a total of 32 hours. As the population grew, the number of CFUs, the number of OMVs, and the amount of PQS in the biofilm all increased over time (Fig 4). We found that peak PQS concentration coincided with the initiation of OMV production, which continued while PQS levels remained high (Fig 4A). As has been reported for planktonic cultures [70], OMV production in biofilms followed PQS accumulation, which helps support a causal link between the two.

### Temporal changes in PA14 biofilm OMV sizes

**Fig 5:**
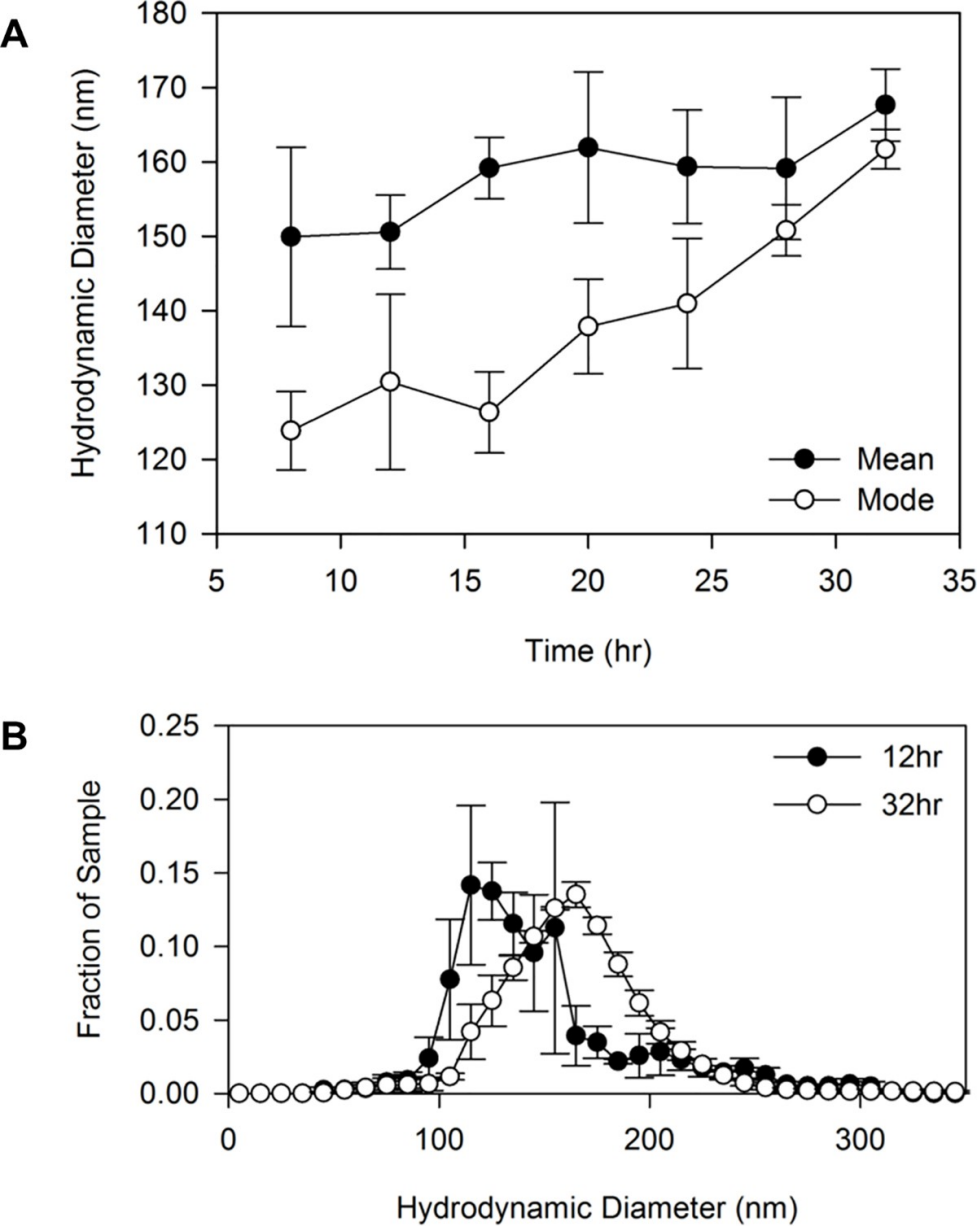
Changes in OMV size over time in PA14 biofilms. The mean and mode sizes of biofilm OMVs harvested every 4 hours were measured by NTA (A). A notable difference in size distribution was found between early stage (12 hours) and late stage (32 hours) biofilms (B). Error bars represent standard error of the mean. n = 3.

As a consequence of using NTA to quantify OMV production every 4 hours, we also obtained data on the size distribution of the OMVs over time. We analyzed whether the size of the OMVs changed as the biofilm developed. A previous dynamic light scattering (DLS) analysis concluded that OMV size was not dependent on the growth phase of planktonic *P*. *aeruginosa* PAO1 cultures [70], though a trend of increasing OMV diameter with time was discernable, albeit not statistically significant. In PA14 biofilms, we observed a progressive change in the OMV size distribution such that, although the mean OMV size differed only slightly as time progressed (trending upward), the mode vesicle size increased significantly (Fig 5A). This is illustrated in an overlay of the 12-hour vs. 32-hour OMV size distributions (Fig 5B). These data suggest that as the biofilm develops, the OMV size distribution shifts to more heavily represent larger vesicles.

### PQS mutant biofilm vesicles are larger and have a wider distribution than wild-type biofilm vesicles

**Fig 6:**
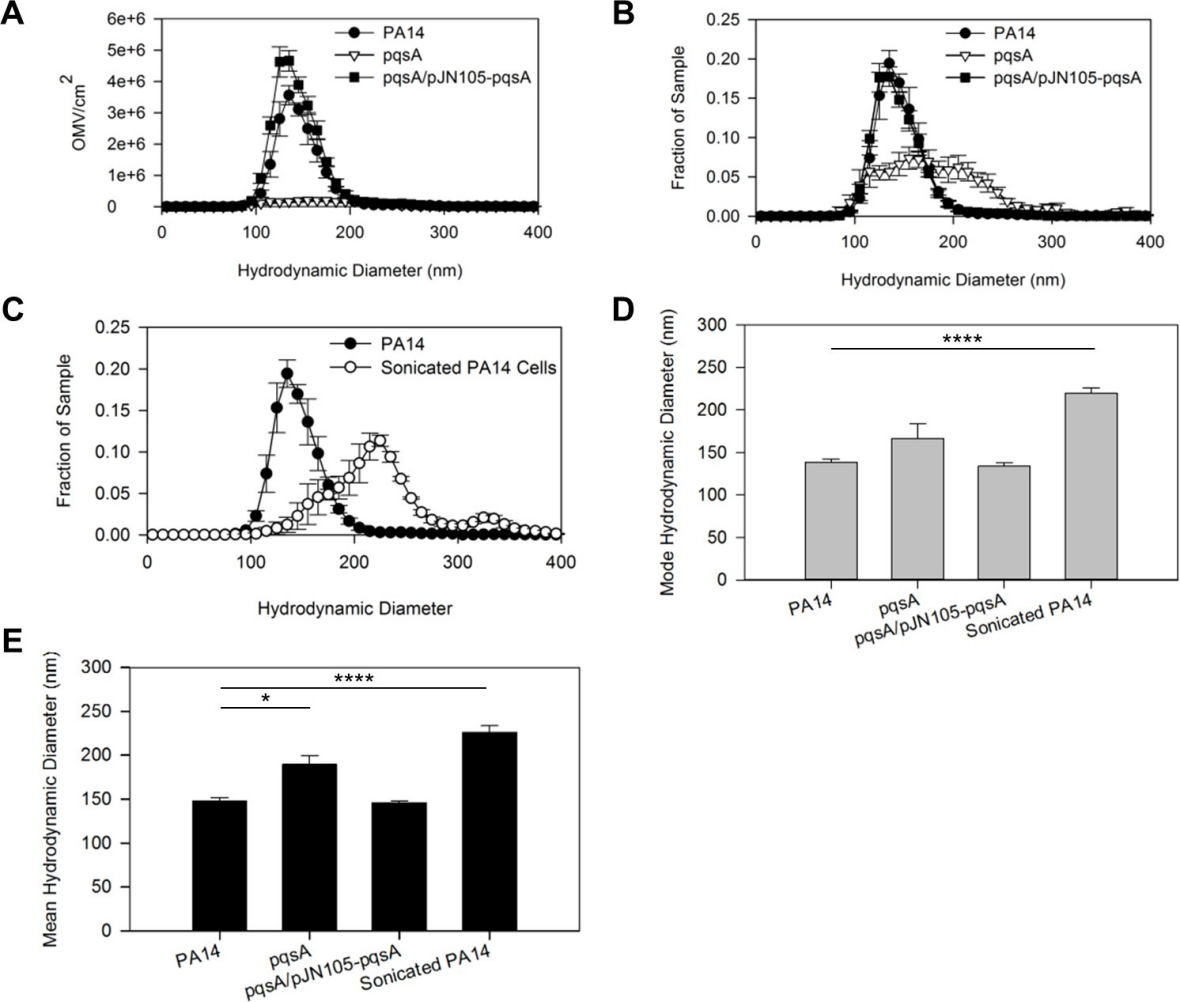
PQS mutant biofilm vesicles are larger and more varied than wild type. Comparison of the size distributions (A-C) and the mode (D) and mean (E) sizes for PA14 OMVs, *pqsA* mutant MVs, complemented *pqsA* mutant OMVs, and sonicated PA14 cell fragments isolated similarly to OMVs. The MV distributions are presented as number of MVs per cm^2^ at each diameter (A), and the relative frequency of MVs at each diameter for each individual sample (B and C) to allow for easier comparison of the distributions. The complemented *pqsA* mutant was grown in the presence of gentamicin at 50 μg/ml. Statistical significance was analyzed by two-tailed Student t-test. Asterisks denote significance relative to the wild-type (* p < .05; ****p < .001). Error bars represent standard error. n≥3.

One possible explanation for the increase in mode vesicle size over time (Fig 5) may be a change in the mechanism of vesicle biogenesis. We hypothesized that a secondary mechanism of vesicle biogenesis that results in the production of large vesicles may become more prominent in later stages of growth in the agar plate biofilm model. We measured a significant reduction in vesicle biogenesis in the *pqsA* mutant, but production was not completely abolished (Fig 3A). We therefore compared vesicle size distributions between the wild type, the *pqsA* mutant, and the *pqsA*-complemented strains to determine whether vesicles produced in the absence of PQS were larger than the wild type and might provide evidence of a parallel mechanism of production. Consistent with the data in Fig 3, this analysis confirmed that the number of OMVs produced by the wild type dwarfed the distribution of vesicles produced by the mutant (Fig 6A). In fact, when plotted as a histogram to allow comparison of vesicles of physiologically-relevant size, the discrepancy between wild type and mutant vesicle production appeared much more stark than when a count of total particles was used (Fig 6A vs. Fig 3A). This apparently larger difference in vesicle production is more in line with both qualitative assessment of ultracentrifuge pellet size (Fig 3B–3E) and previous analyses of PQS-independent vesicle production using biochemical analyses rather than particle counting [19,46,59]. This analysis suggests that the 5-fold difference between vesicle production in the wild type vs. *pqsA* mutant shown in Fig 3 likely represents a conservative estimate of this difference.

It is likely that the minor population of vesicles produced by the mutant is also produced by the wild type, but that the PQS-independent vesicles are obscured by the much greater population of PQS-dependent vesicles produced when PQS is produced. To better compare vesicle sizes, we normalized the distributions to the total number of vesicles produced under each condition and found that the size distribution of *pqsA* mutant vesicles was much wider than wild type vesicles (Fig 6B). Additionally, the distributions for both wild type and mutant had a similar minimum boundary around 85-95nm (Fig 6B), suggesting a possible minimum vesicle size in agar plate biofilms. When *pqsA* was reintroduced to the mutant strain, we found that the OMV distribution matched the distribution of the wild type (Fig 6A and 6B). Taken together, these results suggest that vesicles produced by *P*. *aeruginosa* incapable of synthesizing PQS are larger and more varied in size than the wild-type, but that a PQS-dependent mechanism is responsible for production of the majority of vesicles in our biofilm model.

We hypothesized that the aberrantly-sized MVs found in the *pqsA* mutant biofilms could be due to cell lysis followed by recircularization of the membranes as proposed by Turnbull *et al*. [40]. However, when we tested for cell lysis in our biofilm MV preps, we found no evidence of inner membrane contamination (S4 Fig). Nevertheless, we asked whether the shift in MV size distribution in the *pqsA* mutant could be explained by lysis of a subpopulation of cells too small to be detectable by our lysis assay. To answer this, we compared the size distribution of ultracentrifuged cell fragments following sonication of PA14 cells to the size distributions of WT and *pqsA* mutant vesicles (Fig 6B and 6C). We hypothesized that if lysed cell fragments yielded vesicles that resembled *pqsA* mutant vesicles more than wild type vesicles, this could indicate that different mechanisms of vesicle biogenesis result in the production of different membrane vesicles (MVs), and that a lysis mechanism may be responsible for the minor population of vesicles produced by the *pqsA* mutant. The lysed cell fragments were notably larger than wild type OMVs, with a population distribution that skewed to higher diameter while having a slightly narrower range than the *pqsA* mutant vesicles (Fig 6B and 6C). Consistent with the overall appearance of the distributions, we found that the mode size of sonicated cell fragments was significantly larger that the mode size of PA14 biofilm vesicles (Fig 6D), and that the average sizes of *pqsA* mutant biofilm MVs and lysed cell fragments were both significantly greater than the average size of the PA14 biofilm OMVs (Fig 6E). Specifically, the *pqsA* mutant MVs had a mode diameter of 166.8 ± 41.6 nm and an average diameter of 189.6 ± 9.8 nm, whereas the lysed cell fragments had a mode diameter of 220.0 ± 5.8 nm and an average diameter of 266.3 ± 7.1 nm (Fig 6D and 6E). These values were compared to the wild type, which had an average OMV diameter of 148.1 ± 3.6 nm and a mode OMV diameter of 138.5 ± 3.8 nm (Fig 6D and 6E). While these results do not prove that explosive cell lysis is responsible for the vesicles produced by the *pqsA* mutant, they do suggest that different mechanisms of biogenesis yield vesicles of different sizes, and that we may be able to predict the mechanism of MV biogenesis by analyzing the size of the MVs produced by a bacterial population.

## Discussion

OMVs assist in toxin delivery [17–25,59], communication [19], horizontal gene transfer [26], immune system evasion [30,31], and have been implicated in biofilm formation by either enhancing biofilm development [11] or preventing bacterial attachment [14] in different species. Previous data also show that OMVs are ubiquitous in *in vitro* and *in vivo* biofilms [10,71]. Despite the fact that biofilm OMVs contain different contents vs planktonic OMVs [10,35–37,72], and the fact that biofilms contribute to chronic virulence in bacterial infections [2], the vast majority of studies have focused on studying the functions and biogenesis of OMVs in planktonic bacteria. To understand how OMVs are made and how they are used by bacteria in natural environments, including at infection sites, we must understand how OMVs are produced and used by bacteria living as a biofilm. With this work, we investigate the physical size, function and mechanisms of biogenesis of OMVs in agar plate model biofilms and compare our findings to what has been reported for bacteria growing planktonically or in other biofilm models. We identify similarities and differences between biofilm and planktonic OMVs, and we provide evidence that multiple mechanisms for OMV biogenesis may operate simultaneously in a biofilm, with each gaining prominence under specific environmental conditions.

The mean sizes for PAO1 biofilm and planktonic OMVs presented in this study differed from those presented previously by Schooling and Beveridge [10], though our entire distributions are captured within the large overall ranges that they reported. In a more recent study, Murphy and coworkers found the size of PAO1 planktonic OMVs to be an average of 88.2 ± 1.0 nm in diameter [72], in close agreement with Schooling and Beveridge (85.76 ± 0.87 nm). Both of these studies analyzed OMV size by transmission electron microscopy (TEM), suggesting that the measurement technique may have contributed to the differences between studies. This idea is supported by the work of Tashiro *et*. *al*., who measured planktonic OMVs from PAO1 grown in LB medium by dynamic light scattering (DLS) [70]. Similar to NTA, this technique measures fully hydrated and unprocessed OMV samples and yielded a larger average OMV diameter of 107.3 ± 12.1 nm. Unfortunately, this study also used a different growth medium (LB vs. TSA in our study and the TEM studies). It is possible that the optical techniques (DLS, NTA) undercount very small vesicles because of the requirement for the particles to scatter light combined with the relatively low refractive index of membrane vesicles [73]. However, the manufacturer claims that the NS300 can reliably detect particles down to 10nm in diameter depending on the sample and system configuration, and this instrument has been adopted as a workhorse in the exosome field (exosomes are generally reported to be smaller than OMVs (40–100 nm) [74]). It is worth noting that neither TEM study reported the mode OMV size (i.e. the most common size defining the peak of the distribution) despite having very broad size distributions. Comparison of OMV sizes across conditions using the mode vesicle diameter, as we do here, would be less susceptible to biasing because of irregularities at the extremes due to poor detectability of tiny particles or contamination with larger cell fragments, for example. As is common with many techniques, care must be taken when making specific comparisons across methodologies, though comparison of trends may still be informative.

While analyzing differences in predation of different OMVs, we noticed that the smaller PA14 OMVs were better inhibitors of *S*. *epidermidis* growth than the larger PAO1 OMVs, and the smaller PA14 planktonic OMVs were more effective than the larger PA14 biofilm OMVs. This suggests that there may be a larger concentration of lytic contents in the small OMVs than the larger OMVs, or that another characteristic of the smaller OMVs might allow them to be more potent in our lysis assay. It is important to note that the lack of a zone of inhibition for the PAO1 OMVs does not mean that these OMVs are incapable of promoting cell lysis. It was previously demonstrated that while PAO1 OMVs are able to degrade *S*. *aureus* peptidoglycan [17], these same OMVs did not produce a zone of inhibition in a disk diffusion assay [18], suggesting that disk-diffusion assays may underreport this function for PAO1 (though this assay has been reported here and elsewhere to work well for PA14 OMVs [19]). Nevertheless, these data support the conclusion that OMV predatory potential differs more between strains than between modes of growth of the same strain. Strikingly, our results also suggest a negative correlation between OMV size and predatory activity.

The difference in size and lytic activity reported here between strains PA14 and PAO1 matches their reported virulence potential. PA14 is generally considered to be a more virulent strain than PAO1, attributed largely to the presence of additional pathogenicity islands [75,76]. Our observation that PA14 planktonic OMVs are more effective at lysing *S*. *epidermidis* than PA14 biofilm OMVs may additionally suggest that the use of OMVs for predation is of greater importance in a planktonic phenotype. As the planktonic phenotype is more associated with acute virulence and the biofilm phenotype is more associated with chronic virulence [77–79] this may suggest that OMVs are particularly important for mediating acute virulence.

We also investigated OMV biogenesis in the agar plate biofilm model. It has previously been demonstrated that PQS is responsible for the vast majority of OMVs produced in planktonic *P*. *aeruginosa* [19,59]. However, it was recently reported using the interstitial biofilm model that a RecA-mediated SOS response associated with prophage endolysin activation was responsible for MV production, and PQS did not play a role in MV biogenesis under those conditions [40]. We reasoned that this discrepancy was likely due to the conditions found in an interstitial biofilm, where bacteria are grown for 4–6 hours in nutrient medium solidified with gellan gum that is sandwiched between a closely-opposed glass slide and cover slip in order to produce a monolayer of cells. We would expect this system to be largely anoxic, thereby explaining the reported absence of PQS-induced MVs since molecular oxygen is required as a substrate for PQS synthesis [59]. This interpretation is supported by the fact that the authors were only able to measure endolysin-mediated MV biogenesis in planktonic cells when they were grown anaerobically; aerobically-grown cells likely swamped the system with PQS-induced OMVs that made the endolysin-mediated phenomenon undetectable [40]. We were therefore interested whether PQS was involved in OMV biogenesis in a biofilm model where oxygen is more readily available, at least to some regions of the biofilm. We analyzed OMV production in PA14 and in a PQS biosynthetic mutant [69], and found that the mutant produced at least 5-fold fewer MVs compared to the wild type. This observation suggests that PQS plays a major role in OMV biogenesis in biofilms that resemble agar plate model biofilms. Our finding that OMV production is dependent on PQS biosynthesis in this biofilm model, together with the previous finding that OMV production is not dependent on PQS biosynthesis in interstitial biofilms [40], highlights the likelihood that vesicle biogenesis depends strongly on the microenvironment of cells, with different mechanisms predominating under different conditions.

To follow up on the idea that multiple mechanisms of MV biogenesis may operate independently–and perhaps simultaneously–in biofilms, we further analyzed the small population of vesicles produced by the *pqsA* mutant. We found that they were larger on average than the wild type, and that the mutant vesicle population also had much wider size distribution. Although the failure to detect the presence of inner membrane enzyme activity in OMV preps strongly suggested that cell lysis was minimal in the agar plate biofilms (and the small population of vesicles detected in the mutant supports this), we sought to compare the *pqsA* mutant vesicle population to a control population that we were sure did arise through cellular disintegration followed by vesicle recircularization. We demonstrated that, while not identical, the *pqsA* mutant MV population heavily skewed towards the shape of the distribution of MVs formed through mechanical disruption of cells (via sonication). Interestingly, the size distribution of MVs reported in interstitial biofilms by Turnbull *et al*. [40] matched very closely our distribution of sonicated cell fragments, suggesting that we may be observing a similar vesicle population in the PQS mutant biofilms.

Our evidence of multiple parallel mechanisms for extracellular vesicle production in biofilms is supported by recent investigations of OMV proteomes. The existence of published OMV proteomes from agar plate biofilms is one reason we chose to use this model. Toyofuku *et*. *al*. [36] identified proteins originating from all subcellular locations in biofilm OMVs and in the total biofilm matrix. When compared, however, purified OMVs were under-represented in cytoplasmic proteins and over-represented in OM proteins vs. the total biofilm matrix. The OMVs were *dramatically* under-represented in cytoplasmic proteins and over-represented in OM proteins when compared to parent biofilm cells. Subsequent work by Couto *et*. *al*. [37] reported the 30 most abundant proteins in biofilm OMVs vs. the total biofilm matrix. While proteins from the total matrix where associated with a wide variety of subcellular origins (including 12 from the cytoplasm), the OMVs contained only 1 cytoplasmic-associated protein. Park *et*. *al*. [35] confirmed the strong under-representation of cytoplasmic proteins in both planktonic and biofilm OMVs and notably highlighted that the low *abundance* of cytoplasmic proteins found in the OMV fraction was contributed by a large *number* of unique proteins identified–exactly the situation one would expect if OMV preps included remnants/debris from a small number of disintegrated cells. It is an important consideration that OMV proteomic data is generated from ensemble preparations (often assumed to be homogeneous), which makes the identification of different OMV sub-populations difficult. Results presented in this work demonstrate that the majority of OMVs produced in agar plate biofilms are PQS-dependent, with a small sub-population of differently-sized vesicles also being present. Since PQS induces production of OMVs [19,59] without autolysis [46,48], we propose that the majority population of biofilm OMVs (that are PQS-induced) lack cytoplasmic contents. The minority population of physically different extracellular vesicles are likely produced by a parallel mechanism that may involve SOS response-mediated cell lysis [40] and therefore would be rich in cytoplasmic contents. Consistent with the work of Turnbull *et*. *al*. [40], we show that production of this minority population is not dependent on PQS.

Despite their well-documented abundance in biofilms, almost all that we know about OMVs has come from studies of planktonic bacteria. The work presented here provides important insight into the physical properties, functions and mechanisms of biogenesis of *P*. *aeruginosa* biofilm OMVs. Size differences between planktonic and biofilm OMVs were variable between strains, with the more virulent PA14 producing smaller vesicles under both modes of growth. Consistent with this, the smaller PA14 planktonic OMVs were more potent killers of *S*. *epidermidis* than either type of larger PAO1 vesicle or even the intermediate sized PA14 biofilm OMVs. We demonstrated that the small molecule PQS plays an important role in biofilm OMV biogenesis in our system, as it also does in planktonic cultures. Interestingly, we observed a small population of unusually large MVs produced by a PQS biosynthetic mutant strain whose size distribution approached that of sonicated cellular debris. Reminiscent of recent reports of MVs formed by explosive cell lysis, this population becomes measurable in cell populations where the more dominant PQS-dependent biogenesis mechanism is blocked or reduced. Together, these results suggest that multiple mechanisms for MV biogenesis exist and may come to prominence under differing environmental conditions. Further investigation into the regulation of these different mechanisms and consequences of producing different MV types will help us to understand toxin delivery during chronic biofilm infections.

## Supporting information

S1 Table Primer sequences. Underlined sequences show recognition sites for restriction endonucleases.
(DOCX)

S1 Fig Liberation of OMVs from biofilms by vortexing or homogenization result in similar OMV size distributions. PA14 biofilms were grown and harvested into saline identically. OMVs were then either liberated from the biofilm via vortexing or using a homogenization procedure developed in this publication. NTA was performed to find the distribution (A) and the concentration (B) of the OMVs harvested from the biofilms. Error bars represent standard error of the mean. n≥3.
(TIF)

S2 Fig Homogenization does not result in cell lysis. Planktonic cells suspended in MV buffer to an OD_600_ of 5 were homogenized for 0s, 5s, 10s, 15s, 20s, or lysed via sonication. After homogenization or lysis, the cultures were tested for presence of SDH (A). Additionally, PAO1 (B) and PA14 (C) biofilms were homogenized for 10s or lysed and then tested for presence of SDH. Absorbance values were normalized so that each trial started at a relative absorbance unit of zero. Error bars represent standard deviation. n = 3.
(TIF)

S3 Fig LPS does not contribute to OMV predatory activity. Disk diffusion assays were performed in triplicate using 250 ng of gentamicin as a positive control, MV buffer as a negative control, the approximate amount of LPS found in the 1x10^8^ OMVs for both PAO1 and PA14 in MV buffer, and a 5-fold greater amount of LPS for both strains (A). Images showing representative zones of clearing are also included (B). Zones of inhibition were only seen in the gentamicin control. Error bars represent standard deviation. n = 3.
(TIF)

S4 Fig Wild type and *pqsA* mutant agar plate model biofilm vesicles do not show evidence of cell lysis. PA14 and *pqsA* mutant biofilms were harvested into ice-cold MV buffer, then vesicles were liberated from biofilms via homogenization and isolated via differential centrifugation. PA14 wild type and *pqsA* mutant vesicles were then tested for presence of SDH. To account for the 5-fold decrease in the number of OMVs produced by the mutant, a 5 times larger volume of *pqsA* OMVs was also tested for SDH. As a positive control, PA14 lawns were sonicated after homogenization, and cell fragments were also isolated via ultracentrifugation. As a negative control, MV buffer was also tested for SDH presence. Absorbance values were normalized so that each trial started at a relative absorbance unit of zero. Error bars represent standard deviation. n≥3.
(TIF)
