# Supplementary material for: Analysis of Pseudomonas aeruginosa biofilm membrane vesicles supports multiple mechanisms of biogenesis
Source: PLoS One. 2019 Feb 14;14(2):e0212275. doi: 10.1371/journal.pone.0212275 (PMC6375607; doi:10.1371/journal.pone.0212275)
Supplement: S1 Table — Underlined sequences show recognition sites for restriction endonucleases. (DOCX) [file pone.0212275.s001.docx]

| **Description** | **Sequence (5’ to 3’)** |
| --- | --- |
| *pqsA* pJN105 For | GTAGTACTGCAGAAGAGGGAACGTTCTGTCATGTCC |
| *pqsA* pJN105 Rev | TACTACGAGCTCTCAACATGCCCGTTCCTCCG |
| pJN105 Seq For | CGGCGTCACACTTTGCTATGC |
| pJN105 Seq Rev | GCATAGCAAAGTGTGACGCCG |
