# Supplementary figures and images for: Analysis of Pseudomonas aeruginosa biofilm membrane vesicles supports multiple mechanisms of biogenesis
Source: PLoS One. 2019 Feb 14;14(2):e0212275. doi: 10.1371/journal.pone.0212275 (PMC6375607; doi:10.1371/journal.pone.0212275)

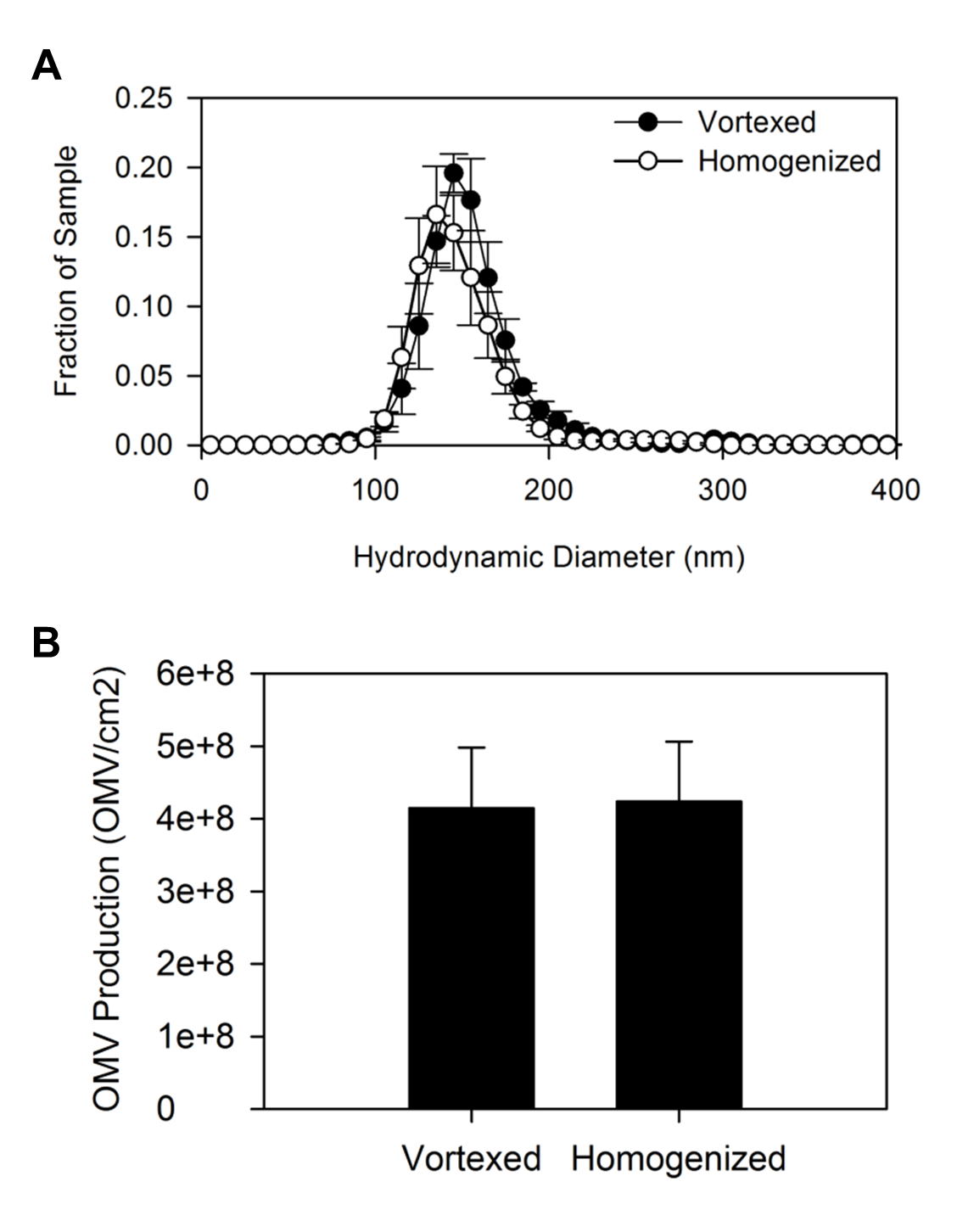

Supplement: S1 Fig — PA14 biofilms were grown and harvested into saline identically. OMVs were then either liberated from the biofilm via vortexing or using a homogenization procedure developed in this publication. NTA was performed to find the distribution (A) and the concentration (B) of the OMVs harvested from the biofilms. Error bars represent standard error of the mean. n≥3. (TIF) [file pone.0212275.s002.TIF]

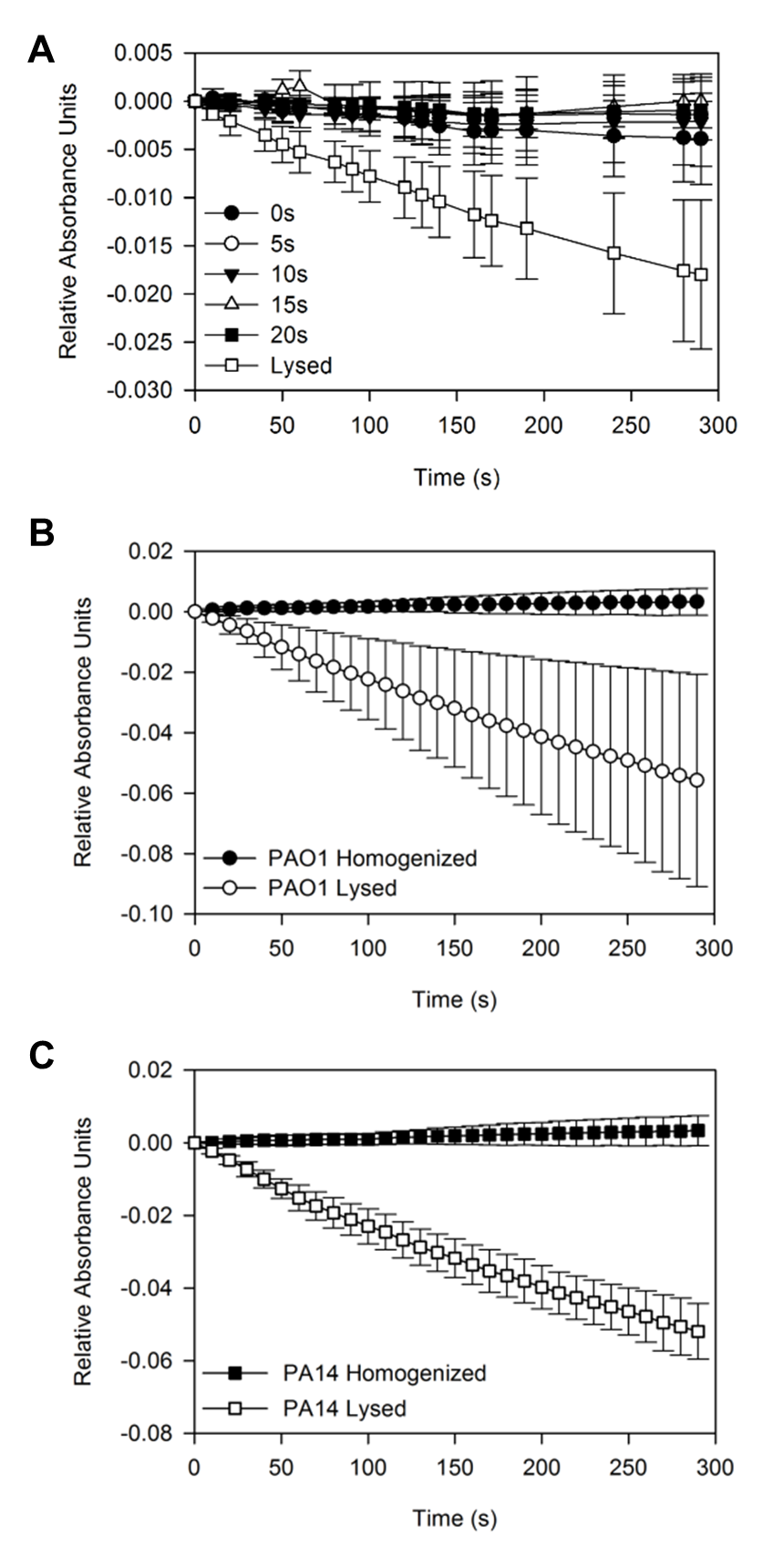

Supplement: S2 Fig — Planktonic cells suspended in MV buffer to an OD600 of 5 were homogenized for 0s, 5s, 10s, 15s, 20s, or lysed via sonication. After homogenization or lysis, the cultures were tested for presence of SDH (A). Additionally, PAO1 (B) and PA14 (C) biofilms were homogenized for 10s or lysed and then tested for presence of SDH. Absorbance values were normalized so that each trial started at a relative absorbance unit of zero. Error bars represent standard deviation. n = 3. (TIF) [file pone.0212275.s003.TIF]

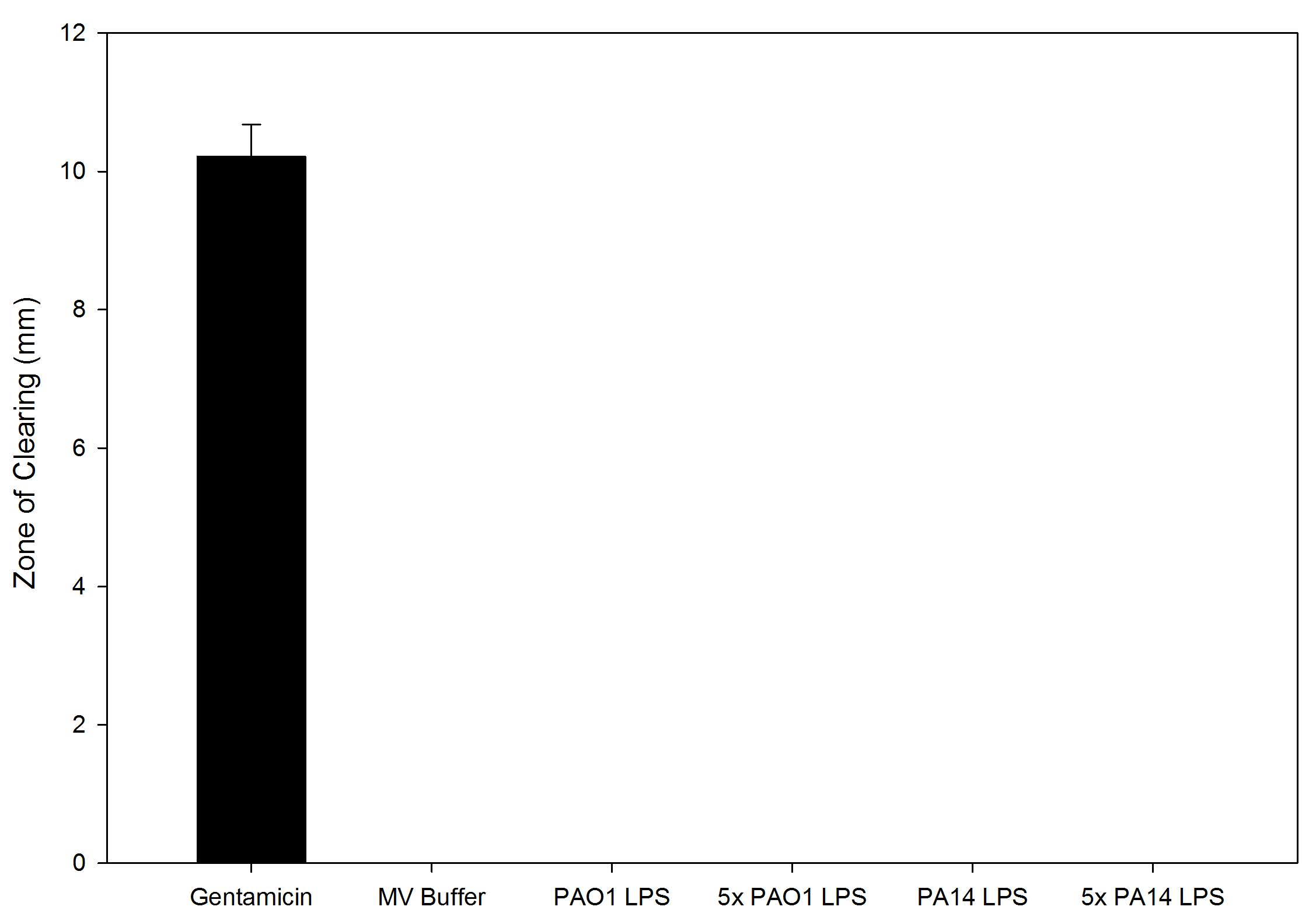

Supplement: S3 Fig — Disk diffusion assays were performed in triplicate using 250 ng of gentamicin as a positive control, MV buffer as a negative control, the approximate amount of LPS found in the 1x10^8^ OMVs for both PAO1 and PA14 in MV buffer, and a 5-fold greater amount of LPS for both strains (A). Images showing representative zones of clearing are also included (B). Zones of inhibition were only seen in the gentamicin control. Error bars represent standard deviation. n = 3. (TIF) [file pone.0212275.s004.tif]

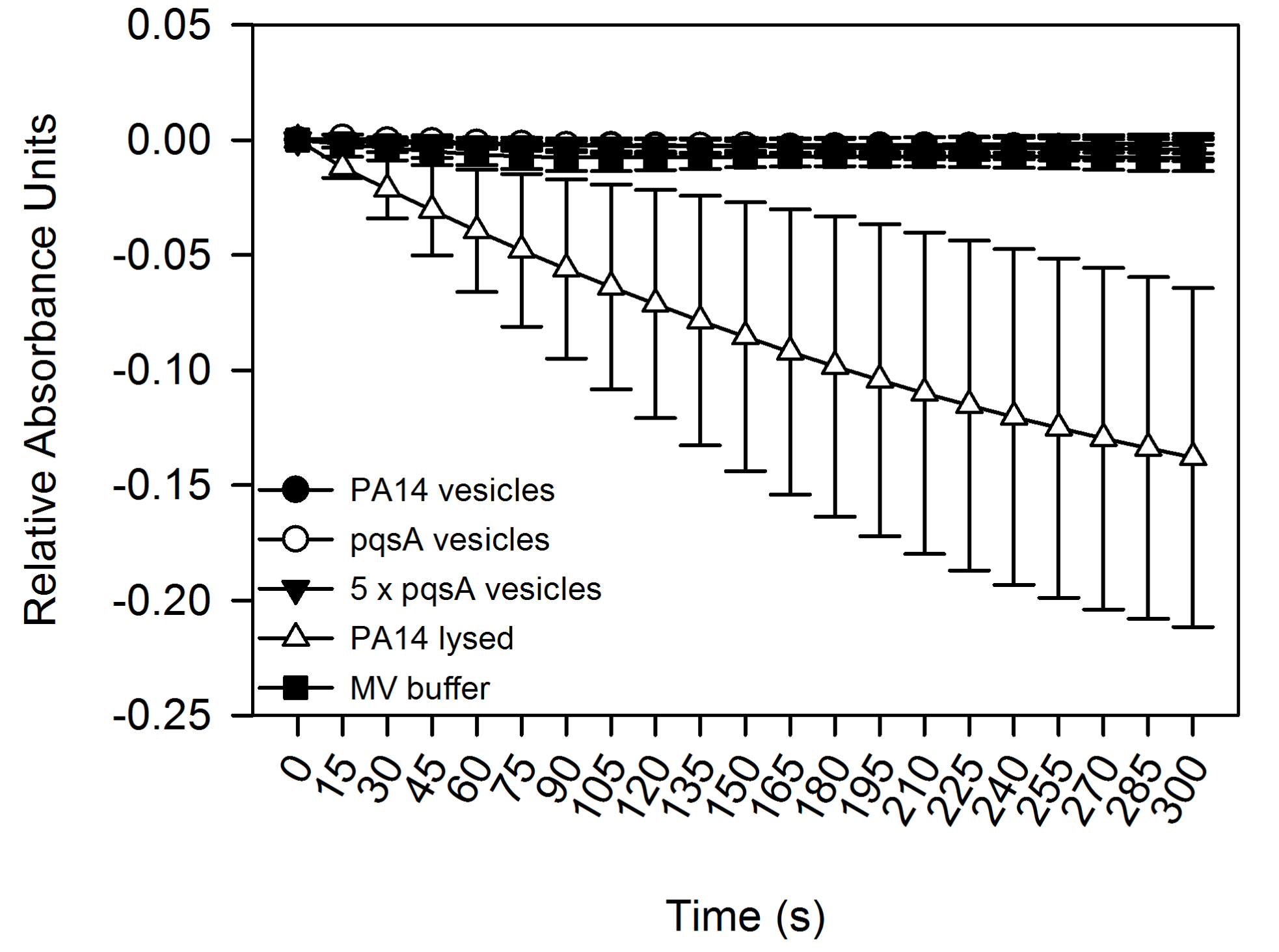

Supplement: S4 Fig — PA14 and pqsA mutant biofilms were harvested into ice-cold MV buffer, then vesicles were liberated from biofilms via homogenization and isolated via differential centrifugation. PA14 wild type and pqsA mutant vesicles were then tested for presence of SDH. To account for the 5-fold decrease in the number of OMVs produced by the mutant, a 5 times larger volume of pqsA OMVs was also tested for SDH. As a positive control, PA14 lawns were sonicated after homogenization, and cell fragments were also isolated via ultracentrifugation. As a negative control, MV buffer was also tested for SDH presence. Absorbance values were normalized so that each trial started at a relative absorbance unit of zero. Error bars represent standard deviation. n≥3. (TIF) [file pone.0212275.s005.tif]
